# Improving detection of familial hypercholesterolaemia in primary care using electronic audit and nurse‐led clinics

**DOI:** 10.1111/jep.12481

**Published:** 2015-11-26

**Authors:** Peter Green, Dermot Neely, Steve E. Humphries, the Medway FH Audit Steering Committee, Tanya Saunders, Val Gray, Louise Gordon, Jules Payne, Slade Carter, Clare Neuwirth, Alan Rees, Hazel Gallagher

**Affiliations:** ^1^ NHS Medway Clinical Commissioning GroupChatham MaritimeKentUK; ^2^ Department of Clinical BiochemistryRoyal Victoria InfirmaryNewcastle upon TyneUK; ^3^ Centre for Cardiovascular GeneticsBritish Heart Foundation LaboratoriesInstitute of Cardiovascular SciencesUniversity College LondonLondonUK; ^4^ Ashfield Healthcare Ltd; ^5^ HEART UK – The Cholesterol Charity; ^6^ MRC Clinical Sciences CentreImperial CollegeLondonUK; ^7^ Cardiff and Vale University Health BoardUK; ^8^ HEART UK patient representative

**Keywords:** audit, clinical decision making, clinical guidelines, diagnosis, familial hypercholesterolaemia, prevention

## Abstract

**Rationale, aims and objectives:**

In the UK fewer than 15% of familial hypercholesterolemia (FH) cases are diagnosed, representing a major gap in coronary heart disease prevention. We wished to support primary care doctors within the Medway Clinical Commissioning Group (CCG) to implement NICE guidance (CG71) and consider the possibility of FH in adults who have raised total cholesterol concentrations, thereby improving the detection of people with FH.

**Methods:**

Utilizing clinical decision support software (Audit+) we developed an FH Audit Tool and implemented a systematic audit of electronic medical records within GP practices, first identifying all patients diagnosed with FH or possible FH and next electronically flagging patients with a recorded total cholesterol of >7.5 mmol L^−1^ or LDL‐C > 4.9 mmol L^−1^ (in adults), for further assessment. After a 2‐year period, a nurse‐led clinic was introduced to screen more intensely for new FH index cases. We evaluated if these interventions increased the prevalence of FH closer to the expected prevalence from epidemiological studies.

**Results:**

The baseline prevalence of FH within Medway CCG was 0.13% (1 in 750 persons). After 2 years, the recorded prevalence of diagnosed FH increased by 0.09% to 0.22% (1 in 450 persons). The nurse advisor programme ran for 9 months (October 2013–July 2014) and during this time, the recorded prevalence of patients diagnosed with FH increased to 0.28% (1 in 357 persons) and the prevalence of patients ‘at risk and unscreened’ reduced from 0.58% to 0.14%.

**Conclusions:**

Our study shows that two simple interventions increased the detection of FH. This systematic yet simple electronic case‐finding programme with nurse‐led review allowed the identification of new index cases, more than doubling the recorded prevalence of detected disease to 1 in 357 (0.28%). This study shows that primary care has an important role in identifying patients with this condition.

## Introduction

Familial hypercholesterolaemia (FH) is an autosomal co‐dominant lipid disorder that confers an increased risk of premature coronary heart disease (CHD) because of lifelong exposure to high concentrations of low‐density lipoprotein‐cholesterol (LDL‐C) [1]. Untreated, FH‐associated elevated LDL‐C results in a greater than 50% risk of CHD in men by the age of 50 years and of at least 30% in women by the age of 60 years [2]. FH is an important, under‐diagnosed cause of premature CHD. National Institute for Health and Care Excellence (NICE) Quality Standard guidance (QS41) [3], European Atherosclerosis Society (EAS) consensus statement [4] and guidance from the International FH Foundation [5] recognize under‐diagnosis of FH as a significant issue to be addressed. The EAS has called for more action for FH as early identification and optimal treatment from a young age is crucial to providing a long and healthy life for affected children and adolescents [6]. In the UK, the Department of Health Cardiovascular Disease Outcomes Strategy recognizes improving identification of inherited cardiac conditions, and FH in particular, as a strategic priority requiring action [7].

According to NICE, the prevalence of heterozygous FH in the UK population is estimated to be 1 in 500, which means that approximately 110 000 people are affected [2]. However, recent population data from Denmark show that possibly up to one in 200 people are heterozygous for FH and one in 160 000–300 000 have homozygous FH [4, 8]. This data are supported by a recent genotyping study conducted in the USA, which demonstrated that 1 in 217 controls carried an *LDLR*‐coding sequencing mutation and had LDL‐C > 4.9 mmol L^−1^ [9]. Based on these data [4, 8, 9], as many as 300 000 people in the UK may have FH. However, a recent national audit estimated that only 15 000 patients have a formal diagnosis of FH [10]. This substantial under‐diagnosis represents a major gap in CHD prevention in the UK, especially when it is proven that reduced exposure to LDL‐C early in life is associated with a large reduction in CHD [11]. Lipid‐lowering treatments, such as statins, can reduce plasma LDL‐C concentrations and reduce mortality in FH patients [12, 13]. Importantly, patients with FH benefit most from early diagnosis and treatment, thus avoiding lifelong exposure to raised LDL‐C with studies showing that diet, lifestyle and statin therapy from a young age substantially delays atherosclerosis progression and reduces cardiovascular risk [6].

FH can be diagnosed using phenotypic criteria and genetic testing. These include very high LDL‐C (>4.9 mmol L^−1^ for adults) on repeat measurements, family history, clinical history of premature CHD and tendon xanthomas and corneal arcus on physical examination. In the UK, the ‘Simon Broome Criteria’ are recommended to evaluate patients with raised LDL‐C [1, 2]. In Europe, the Dutch Lipid Clinic Network (DLCN) criteria are widely used and provide a numerical score to predict the probability of diagnosing FH on genetic testing [4]. This scoring system is increasingly accepted as simple and comprehensive [5], categorizing patients as having definite, probable or possible FH [5].

Genetic testing of FH index cases is recommended and in those cases where a pathogenic mutation is identified systematic cascade testing of close relatives who carry a 50% risk of the disorder, is a cost‐effective approach to diagnose new cases of FH, particularly in younger family members [14]. However, cascade testing from known index cases will not identify all FH cases and fewer than 50% of all cases in a population may be identified by this approach [2]. Therefore, a strategy to detect new index cases is essential to improve diagnosis of FH and prevent CHD within a population [15, 16].

General practitioners (GPs) are well placed to lead on the identification of new index cases – the majority of LDL‐C measurements are requested within primary care and GPs have access to electronic patient records that lend themselves to simple electronic prompts and audits of patient data. The primary care approach to FH diagnosis can be opportunistic, relying on GPs to consider the diagnosis of FH in patients with elevated LDL‐C, or systematic, using an informatics‐based approach to search electronic patient records and identify people who fulfil the profile of FH for further review [16, 17].

In 2008, NICE issued guidance for FH (CG71) recommending that health care professionals consider the possibility of FH in adults with raised cholesterol, especially in those with a personal or family history of CHD [2]. However, despite these guidelines, and subsequent NICE quality standards (QS41) issued in 2013 [3], substantial improvements in FH diagnosis were not being made. NHS Medway CCG considered that increased detection of patients with FH in primary care and the identification of additional ‘at risk’ relatives would lead to better patient management with significant benefit to patients and economic benefit to the CCG through reduced preventable cardiovascular events. NHS Medway CCG set out to support GPs to follow CG71 and introduced a systematic informatics‐based audit of electronic medical records to improve the identification of people with FH followed by a nurse‐led clinic to screen more intensely for new FH index cases.

## Methods

### Setting

This study was conducted in NHS Medway CCG between October 2011 and July 2014. Medway is a CCG of 56 General Practices serving approximately 290 000 patients. Practice sizes range from 1499 to 19 818 people. In 2011, 37 200 people were aged >65 years and 4400 aged >85 years. The ethnic profile of east Kent shows that the population is predominantly white (93.4%, with 2.6% Asian, 1.7% black, 1.4% mixed and 1% Chinese/other). Life expectancy in Medway is significantly worse than the average in England. Medway is ranked within the 41% most deprived boroughs nationally in The Index of Deprivation 2010 and includes an area ranked in the most deprived 3% of areas nationally. This study aligned with the overall vision of NHS Medway CCG – ‘from reactive to proactive for a healthier Medway’ – and with the strategic objective of disease prevention – ‘to prevent people becoming ill and to support people to live healthy and well through a systematic approach in primary care that identifies patients at risk’.

### Development of the FH Audit Tool using electronic audit software

This study utilized Audit+ software (BMJ), a cross‐platform, primary care data analysis tool that was designed specifically for use within consortia, CCGs and other commissioning organizations. Audit+ enables practices to manage their patient registers as defined in an audit specification, easily identify patients who require attention and set the software to prompt the clinician for intervention opportunities automatically when they consult a patient. Audit+ is used widely across NHS Medway CCG, with around 30 audits currently running. As practices are familiar with performing standard audits, no additional training for GPs was required.

The FH Audit Tool was developed in accordance with the Royal College of GPs’ standard criteria for audits and was based on the recommendations of NICE CG71. Specifically, a diagnosis of FH was based on the Simon Broome criteria [2], which classify the patient as definite FH or possible FH (Table 1). As there was no Read Code available for patients classified as ‘possible FH’, we sought and obtained a Read Code from the NHS in 2010. The FH Audit Tool provided prompts to consider a diagnosis of FH based on the Simon Broome criteria (definite or possible FH). The triggers and prompts within the FH Audit Tool are detailed in Table 2. The FH Audit Tool was developed and piloted in a single practice in September 2011 to test and optimize performance. The baseline prevalence of FH was determined at study initiation, defined as all patients previously assigned a Read code for FH according to Simon Broome criteria (Table 2). Improvements in FH diagnosis were assessed over the 2‐year time period.

**Table 1:** The Simon Broome Register criteria (total cholesterol and LDL‐C levels either pre‐treatment or highest on treatment) [1, 2]

Definite FH	Possible FH
Total cholesterol >6.7 mmol L^−1^ or LDL‐C > 4.0 mmol L^−1^ in a child aged <16 years or Total cholesterol >7.5 mmol L^−1^ or LDL‐C > 4.9 mmol L^−1^ in an adult	Total cholesterol >6.7 mmol L^−1^ or LDL‐C > 4.0 mmol L^−1^ in a child aged <16 years or Total cholesterol >7.5 mmol L^−1^ or LDL‐C > 4.9 mmol L^−1^ in an adult

Plus	And at least one of the following
Tendon xanthomas in the patient or a first‐degree (parent, sibling or child) or second‐degree relative (grandparent, uncle or aunt) or DNA‐based evidence of an LDLR mutation, familial defective APOB‐100 or a PCSK9 mutation	A family history of myocardial infarction: <50 years of age in second‐degree relative or <60 years of age in first‐degree relative or A family history of raised total cholesterol: > 7.5 mmol L^−1^ in an adult first‐degree or second‐degree relative or >6.7 mmol L^−1^ in a child or sibling aged <16 years

**Table 2:** Triggers and prompts provided by FH Audit Tool

Trigger	Prompt
Patients with FH or possible FH whose family has not been informed	Have relatives been informed regarding FH?
Patients with FH, possible FH or probable FH whose latest total cholesterol is >5 mmol L^−1^	Up‐titrate statins or consider referral*
Patients whose latest cholesterol is >7.5 mmol L^−1^ or LDL‐C > 4.9 mmol L^−1^ and who have had a positive genotype test	Diagnose FH
Patients whose latest cholesterol is >7.5 mmol L^−1^ or LDL‐C > 4.9 mmol L^−1^ and have a family history of premature CHD and/or hypercholesterolaemia and have not had a Simon Broome assessment	Consider possible FH
Patients whose latest cholesterol is >7.5 mmol L^−1^ or LDL‐C > 4.9 mmol L^−1^, have not had a Simon Broome assessment and have a family history of CHD but no details of the age of the relatives	Ask patient if myocardial infarction has occurred before 50 years of age in a second‐degree relative or before 60 years of age in a first‐degree relative – Yes, then consider FH; No, assess using Simon Broome criteria

*Assessment of this prompt was not undertaken as part of this audit.

The nurse‐led clinics conducted in the second part of the study employed the Simon Broome Criteria as well as the DLCN criteria (Table 3). This alternative phenotypic scoring system for FH provides a numeric score and defines FH as definite, probable and possible. Prior to the introduction of nurse‐led clinics, a read code for probable FH was requested and provided and the FH Audit Tool enhanced to include the DLCN score to define the severity of FH to further support clinical management.

**Table 3:** Dutch Lipid Clinic Network criteria for making a phenotypic diagnosis of FH in adults [4]

Criteria	Score
Family history	
First‐degree relative with known premature coronary and/or vascular disease (men aged <55 years, women aged <60 years) or First‐degree relative with known LDL‐C above the 95th percentile for age and gender	1
First degree relative with tendinous xanthomata and/or arcus cornealis or Children aged <18 years with LDL‐C above the 95th percentile for age and gender	2
Clinical history	
Patients with premature coronary artery disease (men aged <55 years, women aged <60 years)	2
Patients with premature cerebral or peripheral vascular disease (men aged <55 years, women aged <60 years)	1
Physical examination	
Tendinous xanthomata	6
Arcus cornealis before 45 years of age	4
Investigation	
LDL‐C ≥ 8.5 mmol L^−1^	8
LDL‐C 6.5–8.4 mmol L^−1^	5
LDL‐C 5.0–6.4 mmol L^−1^	3
LDL‐C 4.0–4.9 mmol L^−1^	1
Diagnosis	Total score*
Definite FH	>8
Probable FH	6–8
Possible FH	3–5
Unlikely FH	<3

*Total score is calculated by adding together the single highest score from each of the four domains.

### Systematic assessment for FH diagnosis

The FH Audit Tool identified and flagged all patients at potential risk of FH according to elevated cholesterol (total cholesterol level >7.5 mmol L^−1^ in adults or >6.7 mmol L^−1^ in children <16 years or LDL‐C > 4.9 mmol L^−1^ in adults or >4.0 mmol L^−1^ in children) for further assessment. These patients were termed ‘at risk and unscreened’. During the course of the next 2 years, electronic prompts appeared when the patient was in the practice that sought to enhance the GPs’ decision making on FH diagnosis and to help confirm or refute an FH diagnosis.

### Enhanced assessment for FH with nurse‐led clinics

The nurse‐led clinics, which formed an ‘FH Nurse Advisor Programme’, began in October 2013 and ran for 9 months to study end. The programme was conducted by one nurse employed by Ashfield Health Care Ltd and trained by HEART UK and care was consistent with the NICE clinical guideline 71 [2]. The nurse was governed by the Nursing and Midwifery Council Code of Professional Conduct.

**Figure 1:**
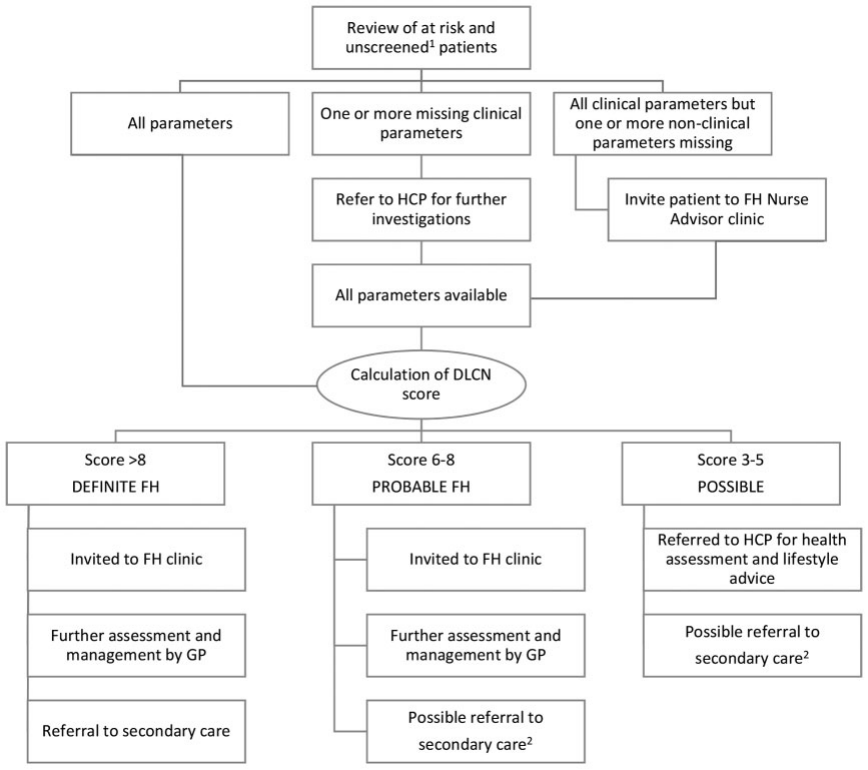
Enhanced assessment for FH with nurse‐led clinics. ^1^ At ‘risk and unscreened’ patients identified via FH Audit Tool, that is, total cholesterol level >7.5 mmol L^−1^ in adults or >6.7 mmol L^−1^ in children <16 years or LDL‐C > 4.9 mmol L^−1^ in adults or >4.0 mmol L^−1^ in children and unscreened by Simon Broome. ^2^ Patients with probable and possible FH were managed within primary care but with secondary care referral if LDL‐C levels were not controlled, if there was a family history of specialist lipid management, or if there was a family history of vascular events.

The nurse reviewed the audit list of ‘at risk and unscreened patients’ to identify any missing clinical or non‐clinical parameters in individual patient records that prevented a calculation of a DLCN score. Missing clinical parameters were sought from the relevant health care professionals and missing non‐clinical parameters, for example, incomplete family history, triggered an invitation to the FH nurse advisor clinic. Once all parameters were collated, a DLNC score was calculated. Patients with DLNC scores ≥6, which indicated definite or probable FH, and those patients with missing non‐clinical parameters, were invited to attend the FH nurse advisor clinic. Those patients with possible FH (score 3–5), were referred to other health care professionals for health assessment and lifestyle advice. This pathway is shown in Fig. 1.

At the clinic, conducted at a patient's own GP surgery, the nurse explained the purpose of the clinic, provided a Patient Service User Guide and an FH education leaflet and obtained written consent. During the 30‐minute appointment, clinical examination for xanthoma or corneal arcus was conducted, and a family history recorded as specified in Simon Broome and DLCN. Cascade testing was discussed and if a diagnosis of FH was made, patients were provided with cascade letters to pass on to their first‐degree relatives. The role of lifestyle factors was discussed and information and advice were offered, aimed at improving patient concordance to prescribed lipid‐lowering medication and increasing the patient's understanding of their disease. The FH Nurse Advisors role was limited to discussing FH, disease severity and any management issues based on clinical assessment and current NICE guidelines. In the event of a patient not attending the clinic, the patient was flagged to the GP practice for appropriate follow‐up.

After clinic completion, the FH Nurse Advisor attended the practice at a later date and worked with the practice to ensure the findings of the FH review were included in the electronic clinical records. Any medical interventions were decided by the GP. The Nurse Advisor made recommendations to the GP based on the clinic review – those patients diagnosed with FH were recommended for referral to secondary care for specialist management; probable or possible FH were recommended for management within primary care, but with secondary care referral if LDL‐C levels were not controlled, if there was previous referral to secondary care of a family member, or if there was a family history of vascular events.

## Results

The majority of GP practices (53/56, 95%) in NHS Medway CCG participated in the Audit. Three practices without Audit+‐compatible IT systems were unable to take part and accounted for approximately 8000 patients (at study close in July 2014). Forty‐seven of 53 GP practices (89%) participated in the FH Nurse Advisor Programme. One practice closed during the nurse advisor programme, and the remaining five GP practices that did not participate in the nurse programme each serviced an average 5000 patients. Despite this, no areas of the CCG were excluded, as the areas of participating practices overlapped with non‐participating practices. The Nurse Advisor Programme reviewed the records of 1505 patients. Of 210 patients invited for clinic visits, 109 (52%) attended.

**Figure 2:**
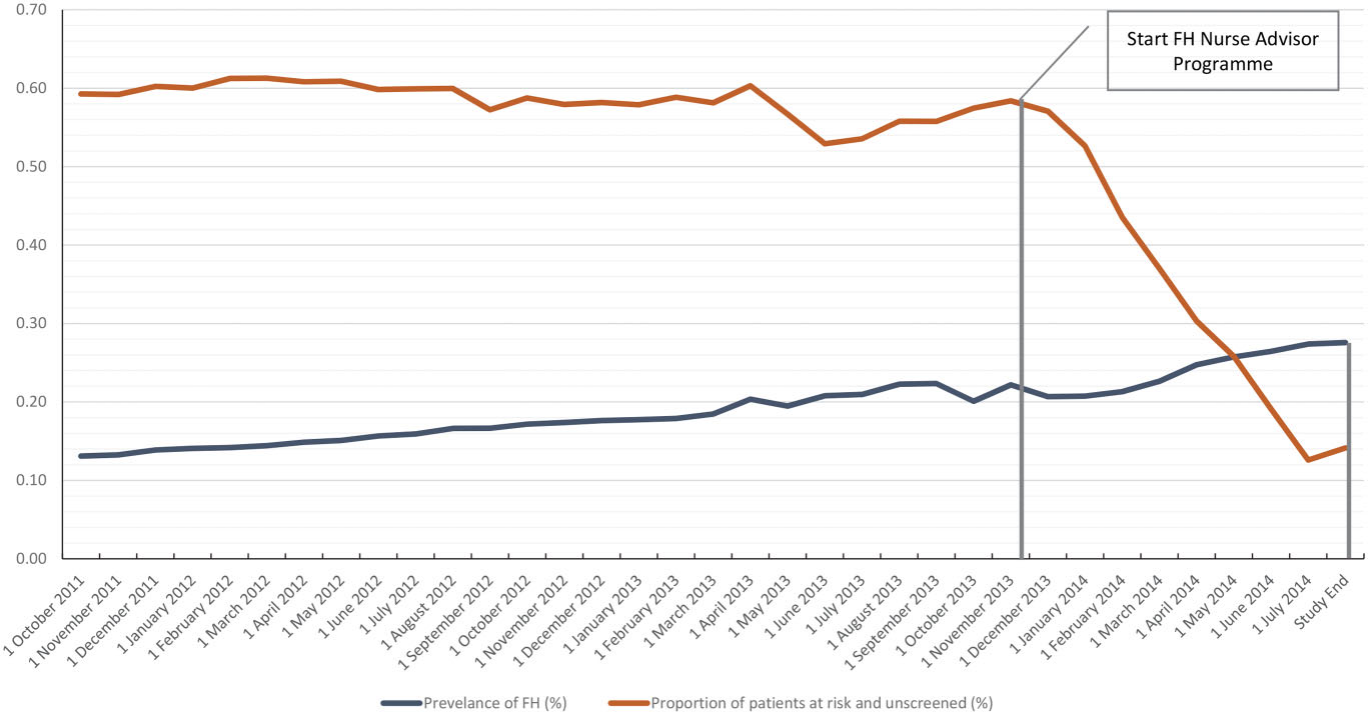
Recorded prevalence of FH diagnosis and proportion of patients ‘at risk and unscreened’ over the course of the study. October 2011–November 2013: all FH diagnoses made by Simon Broome criteria, after November 2013 FH diagnoses made by Simon Broome criteria and/or DLCN score; patients were considered to be ‘at risk and unscreened’ if they had a total cholesterol >7.5 mmol L^−1^ and/or LDL‐C > 4.9 mmol L^−1^ (adults) and had not been assessed using the Simon Broome criteria.

The baseline recorded prevalence of FH within Medway CCG was 0.13% (1 in 750 persons). The proportion of patients ‘at risk and unscreened’ was 0.59% (Fig. 2; Table 4). After 2 years, the recorded prevalence of diagnosed FH increased by 0.09% to 0.22% (1 in 450 persons). The proportion of patients ‘at risk and unscreened’ was 0.58%. After the completion of the Nurse Advisor Programme, the recorded prevalence of patients diagnosed with FH increased to 0.28% (1 in 357 persons). This included 0.03% of patients that were categorized as ‘probable FH’ based on the DLCN score. The proportion of patients ‘at risk and unscreened’ was reduced to 0.14%, and this reduction is shown in Fig. 2.

**Table 4:** Medway FH Audit Tool results

	Baseline^a^ (October 2011)	2‐year audit^a^ (October 2013)	FH Nurse Advisor Programme^b^ (July 2014)
Population	262 030	199 346 c	281 655

	Number	Prevalence (%)	Number	Prevalence (%)	Number	Prevalence (%)
FH	331	0.13	354	0.18	546	0.19
Probable FH	NA^d^	NA^d^	NA^d^	NA^d^	83	0.03
Possible FH	12	0.005	88	0.04	147	0.05
Total FH	343	0.13	442	0.22	776	0.28
At risk and unscreened	1553	0.59	1164	0.58	398	0.14

a FH diagnoses made by Simon Broome criteria; patients were considered to be ‘at risk and unscreened’ if they had a total cholesterol >7.5 mmol L^−1^ and/or LDL‐C > 4.9 mmol L^−1^ and had not been assessed using the Simon Broome criteria.

b FH diagnoses made by Simon Broome criteria and/or DLCN score.

c Population (and number of FH) is lower than previous time‐point; data could not be extracted from all electronic medical information systems at this time.

d FH diagnoses made by Simon Broome criteria, which does not include ‘probable FH’.

NA, not applicable.

In terms of patient numbers, an additional 99 cases of FH were diagnosed during the 2‐year period. During the 9 months of the nurse advisor programme, an additional 334 cases were diagnosed – 192 with definite FH (DLNCS >8), 83 patients with probable FH (DLCNS 6–8) and 59 with possible FH (DLNCS 3–5). Overall, an additional 433 index patients with FH were identified within NHS Medway CCG (Table 4). The number of people at risk and unscreened reduced from 1553 to 398 and this reduction is seen in Fig. 2.

## Discussion

Our study shows that two interventions increased the detection of FH within a CCG. Using an electronic audit tool, running on GP electronic patient record systems increased the recorded prevalence of diagnosed FH from one in 750 at audit initiation (0.13%) to one in 450 (0.22%) after 2 years. Opportunistic identification of patients with specific computer reminders had little impact on the number of patients identified as at risk and unscreened. The two‐stage process of systematic identification with FH nurse specialist assessment increased the proportion of patients diagnosed with FH to one in 357 (0.28%), approaching the Danish and US estimated FH population prevalence of approximately one in 200 [4, 8, 9]. The programme identified 433 new FH index cases, more than doubling the prevalence of detected disease and allowing appropriate referral to secondary care for cascade testing. We suggest the burden placed on individual practices by this increase in FH diagnosis is manageable. For a large practice of more than 10 000 patients, approximately 60 patients would require further review. After assessment, our prevalence figures suggest about 30 new index cases would be identified and require referral and ongoing management.

GP participation in this study was high. The FH Audit Tool was incorporated in the majority of GP practices in Medway (95%) and all practices with compatible electronic patient record systems participated. Eighty‐nine per cent of eligible practices took part in the Nurse Advisor Programme with six eligible practices declining to participate, but the audit tool remained in use. The full geographical area of the CCG was covered, with the practice areas of participating practices overlapping with non‐participating practices. The proportion of patients accepting the invitation to the nurse clinic was high (52%). A further strength of the study is the timeframe, with FH prevalence recorded over an almost 3‐year period.

An obvious limitation was the completeness of the electronic patient records. The FH nurse sought missing data from the health care professional or patient, but undocumented information may have resulted in missed diagnoses. This is particularly pertinent for family history of CHD, with patients perhaps unaware of the extent of CHD in their family or where such information had not been collected or recorded. This was a pragmatic clinical audit conducted in real‐world practice. As such, there was no retrospective review of patients previously coded as FH, either at baseline or after the introduction of DLNC to confirm the previous Simon Broome FH diagnoses. Coding in primary care is notoriously inaccurate, and therefore, the baseline prevalence reported may not be accurate.

Identification of FH via the FH Audit Tool relies on a record of total cholesterol and or LDL‐C values in patients' electronic record. Patients aged 40 years and above are more likely to have lipid values recorded as a consequence of the NHS Health Check programme. Younger patients, in particular children and young adults, are unlikely to have lipid values on record. This points to the importance of identifying new index cases, which can then trigger cascade testing to identify younger family members who may not be identified by an electronic audit. It is unknown what proportion of the Medway population had lipid results available for assessment in this study.

NICE guidance recommends genetic testing of all index cases and cascade testing of family members as a cost‐effective method for identifying new cases of FH [2]. Genetic testing for FH is not routinely available in England and was not included as part of this audit. However, with the new NHS commissioning structure and its commitment to increased investment into genetic sequencing resources, genetic confirmation of clinically diagnosed FH cases is certainly feasible [18, 19]. When available, genetic testing will allow mutation carriers to be distinguished from those with polygenic FH [19] and focus resources on cascade testing in the 40% of clinically diagnosed FH patients with an identified single gene alteration [15].

The purpose of the audit was to increase detection of FH and suggest appropriate management of these patients based on NICE clinical guidance [2]. However, the FH audit tool did not allow assessment of pre‐ and post‐diagnosis lipid levels; therefore, in the context of this study, it is not known if diagnosis of FH led to improved patient management and a reduction in lipid levels. A future FH Audit Tool should address this issue and potentially measure changes in lipid levels pre‐ and post‐diagnosis to assess patient management. Secondary causes of raised lipids are an important clinical consideration. Secondary causes of raised lipids could be assessed in patients ‘unscreened and at risk’ and invited to the nurse clinic. Future programmes should consider a clinic visit for all unscreened and at‐risk patients to eliminate secondary causes of dyslipidaemia.

Our study demonstrates that there is an opportunity to increase the diagnosis of FH by exploiting the information contained within GP electronic patient records using automated systems. While each new diagnosis allows appropriate management and treatment of that person, an additional benefit is that each new case acts as a trigger for cascade testing to identify younger family members who may not otherwise be detected and who benefit most from early diagnosis and appropriate treatment. Ultimately improving diagnosis should lead to optimized therapeutic intervention and hence reductions in CHD in this high‐risk patient group. The next step is to enhance the FH Audit Tool to measure the impact of diagnosis on patient management and to provide the FH Audit Tool widely to other interested CCGs.

## Conflict of interest

All authors have completed the Unified Competing Interest form at http://www.icmje.org/coi_disclosure.pdf (available on request from the corresponding author) and declare that PG has received personal fees from BMJ, outside the submitted work. DN has received honoraria for participation in advisory boards for Genzyme, Roche, Aegerion, Sanofi and Amgen. DN has received sponsorship from MSD to attend an educational meeting. DN has been Newcastle upon Tyne hospital NHS Foundation Trust site investigator for clinical trials sponsored by KaraBio, MSD, Sanofi, Amgen and ISIS. DN is a Trustee and board member of HEART UK – the Cholesterol Charity and Co‐Chairman of the Familial Hypercholesterolemia Guideline implementation group and is a member of Newcastle FATS guideline group on lipid modifying treatment.

## Author contributions

Contributors: PG conceived and designed the audit design, with additional support from SEH and DN. PG was involved in data cleaning. PG, SEH and DN analysed the data. PG, SEH and DN wrote the first draft of the manuscript, which was then critically reviewed and revised by the other co‐authors. PG, SEH and DN are members of the Medway FH Steering committee. All authors and members of the Steering committee approved the final version of the manuscript for submission. All authors had full access to all of the data (including statistical reports and tables) in the study and can take responsibility for the integrity of the data and the accuracy of the data analysis.
